# Engaging fathers to support child nutrition increases frequency of children’s animal source food consumption in Rwanda

**DOI:** 10.1371/journal.pone.0283813

**Published:** 2023-04-07

**Authors:** Valerie L. Flax, Emily A. Ouma, Mary-Ann Schreiner, Adeline Ufitinema, Eugene Niyonzima, Kathleen E. Colverson, Alessandra Galiè

**Affiliations:** 1 RTI International, Research Triangle Park, North Carolina, United States of America; 2 International Livestock Research Institute, Kampala, Uganda; 3 Three Stones International, Kigali, Rwanda; 4 National Child Development Agency, Ministry of Gender and Family Promotion, Kigali, Rwanda; 5 Department of Food Science and Technology, College of Agriculture, Animal Science and Veterinary Medicine, University of Rwanda, Musanze, Rwanda; 6 Division of Animal Resources Processing & Biotechnology, Rwanda Agriculture and Animal Resources Development Board, Huye, Rwanda; 7 Department of Animal Sciences, University of Florida, Gainesville, Florida, United States of America; 8 International Livestock Research Institute, Nairobi, Kenya; UNITED STATES

## Abstract

Although social support from fathers is associated with improved child feeding practices, evidence on feasible, acceptable, and effective ways to involve fathers in supporting child nutrition, including animal source food (ASF) consumption, is limited. This study was a follow-on to a trial that tested the effects of social and behavior change communication (SBCC) targeted mainly at mothers to promote ASF consumption by children in households that received an exotic or crossbred cow through the government of Rwanda’s Girinka “One Cow Per Poor Family” program (NCT0345567). A delayed SBCC intervention was provided to mothers in the non-intervention arms prior to the present pre/post study, which targeted fathers in households across the trial study arms. Baseline and endline surveys with a cohort of 149 fathers with a child <5 years were used to evaluate the effects of a SBCC intervention for fathers on their children’s ASF consumption and on fathers’ knowledge, awareness, and support for children’s ASF consumption. Qualitative data collected from fathers, mothers, and program implementers were used to assess feasibility and acceptability of the intervention for fathers. The SBCC intervention comprised group meetings led by model fathers, text messages, print materials, and megaphone blasts. The odds of children consuming any type of ASF ≥2 times in the last week increased from baseline to endline (OR 4.9, 95% CI 1.9, 12.3), as did the odds consuming milk, eggs, and beef, but not fish. Fathers’ mean ASF knowledge and awareness scores increased from baseline to endline (knowledge: 2.3 to 3.5 out of 4 items, *P*<0.001; awareness: 2.5 to 3.0 out of 3 items, *P*<0.001), with the largest changes observed in knowledge of timing of introduction of milk and other ASFs. The percentage of fathers who offered two or more supportive actions for their children’s milk and other ASF consumption increased from baseline to endline (milk: 19.5% to 31.5%, *P* = 0.017; other ASFs: 18.8% to 37.6%, *P*<0.001). Fathers appreciated gaining knowledge on child nutrition in a setting specifically for men and liked the print materials that offered clear actions they could take to support their children’s ASF consumption. This study shows that an SBCC intervention for fathers can improve children’s ASF consumption and increase fathers’ knowledge, awareness, and support for children’s nutrition.

## Introduction

Animal source foods (ASFs) provide essential nutrients and are important for child growth and development [1, 2]. Consumption of ASFs and other optimal child nutrition practices are often influenced by gender inequality and gender roles that impact household decision-making and provision of social support [3–5]. In many low- and middle-income countries (LMICs), cultural norms indicate that women are responsible for childcare and feeding, while men are responsible for providing resources for and leading the family [6–8]. These gender roles constrain women’s ability to carry out optimal child feeding practices, including adequate feeding of milk and other ASFs [9], because men are expected to provide ASFs and women often have limited decision-making power and resources to procure them, if men do not fulfil their role [10]. Men can positively or negatively influence child feeding and household nutrition in several ways, including through their decisions about which crops to plant or livestock to raise, how crops and livestock are managed, how to use money they and their wives earn, whether to keep or sell food produced within the household, and which types of food to buy [11].

In Rwanda prior to the 1994 genocide, traditional gender roles for women included childcare and food preparation. A gender analysis study showed that cultural norms limited women’s ability to work outside the home without their husbands’ permission [12]. Men controlled household finances and had final decision-making power. After the genocide, the government enacted policies and quotas to improve gender equality in politics and other aspects of life [13, 14]. Women now have the right to inherit and own land, be elected to official positions, speak on behalf of the family, and work outside the home. Decision-making and sharing in earning money and supporting the household is now more equal in Rwanda than in the past. However, results from a recent qualitative analysis showed that men still have final decision-making power in households, and women still perform traditional roles, like food preparation, childcare, and child feeding [15]. Women report that decisions about use of food (e.g., milk) from animals owned by the household are often made jointly by women and men [16]. However, men have more money and agency than women, which puts them in control of decisions about purchasing and consumption of ASFs procured outside the household [17].

Studies in LMICs have shown improvements in child feeding practices and nutritional outcomes when fathers are involved in decision-making on young child feeding and when they provide physical, psychological, and financial support for child feeding practices [18, 19]. The majority of child feeding and nutrition interventions that included fathers reached out to them as families or couples, often engaging fathers only if they happened to be present [20]. Several studies have specifically developed or tailored strategies for reaching or engaging men in child feeding [6, 19, 21–27], but only a few of these have quantitatively measured changes in men’s knowledge, attitudes, and social support [19, 22].

Despite the role of gender dynamics and norms and the importance of fathers in influencing nutrition in their households, the evidence on feasible, effective, and acceptable ways to involve fathers in child nutrition is limited [9, 20]. To address this gap, we designed and implemented a social and behavior change communication (SBCC) strategy to engage fathers in understanding and supporting child nutrition practices, especially consumption of milk and other ASFs. The aims of this study were to (1) evaluate the effects of an SBCC intervention for fathers on children’s ASF consumption and on fathers’ knowledge, awareness, and support for ASF consumption by their young children and (2) collect data on the feasibility and acceptability of the intervention from participants and implementers.

## Methods

### Study overview

This was a follow-on study to a trial that tested the effects of an SBCC intervention promoting ASF consumption by children in households that received an exotic or crossbred cow through the Government of Rwanda’s Girinka “One Cow Per Poor Family” program (NCT0345567) [28]. The trial was conducted in Nyabihu and Ruhango Districts from 2018 to 2020 and provided SBCC to mothers but included fathers when available. The trial showed that the SBCC intervention increased mothers’ knowledge and awareness of ASF consumption by children, and children’s odds of consuming milk two or more times in the last week increased with maternal exposure to the intervention [28]. A delayed intervention for mothers was implemented in the non-invention arms of the trial prior to beginning the present study.

The follow-on study used a pre/post evaluation design because the trial (referred to henceforth as the initial study) had already enrolled all Girinka and Girinka-eligible households with a child in the target age range in the two districts and provided an ASF SBCC intervention to the women in those households. Therefore, no comparable control group for the follow-on within the districts was available. The follow-on study was conducted from 2020 to 2021 and targeted ASF-related SBCC specifically to fathers. It included men from all study arms in the initial study. Three Stones International, in collaboration with the National Child Development Agency and the Rwanda Biomedical Center, designed and implemented the SBCC intervention encouraging men to support milk and other ASF consumption by their children.

The intervention was evaluated by the International Livestock Research Institute (ILRI), RTI International, and the University of Rwanda using baseline and endline surveys with a cohort of fathers from the initial study. Qualitative data were collected from fathers, mothers, and program implementers at endline to gather information about the feasibility and acceptability of the SBCC intervention.

### Intervention

Intervention design was based on findings from the initial study plus additional formative research with men, women, and key informants conducted as part of the follow-on study to learn about the barriers to and enablers of men’s participation in nutrition and the best channels to reach them [28, 29]. The University of Florida used the formative data to develop and conduct a 3-day training for Three Stones International and the National Child Development Agency on promising methods to engage fathers more actively in household nutrition. The training was used to identify intervention modalities (i.e., who should deliver the intervention, times and locations for intervention activities) and was adapted for use in training implementers to carry out the intervention. The 5-month intervention was implemented from June through October 2021, but no in-person activities were permitted in July and August 2021 because of government COVID restrictions on travel and gatherings.

The formative research was also used to develop key messages (**S1 File**) and select communication channels, which included group meetings for men, weekly text messages to men’s mobile phones, and megaphone blasts. The group meetings were targeted at fathers, but mothers could also participate. They were held in each community once per month (but not in July and August) at a variety of venues where fathers are commonly present, including sector or cell offices, savings groups meetings, parent evenings, community meetings and gatherings, after church community announcements, weekly community assemblies, and cooperative meetings. Supporting materials included a leaflet distributed to fathers and a poster and megaphone blasts used during group meetings.

The group meetings were led by 29 community extension and health officers (CEHOs) and 23 model fathers, who were trained by the project. Model fathers were selected from intervention households in each administrative sector. They were identified by CEHOs and agriculture extension workers as leaders in their communities with some knowledge on nutrition and animal husbandry. CEHOs and model fathers were paid 5,000 Rwanda francs (approximately 5 US dollars) per month to conduct one group meeting monthly to share key messages with fathers, collect accurate phone numbers for men in the cohort, distribute the leaflet to men in the cohort, attend one meeting with the Three Stones coordinators, and submit a monthly report of their activities. CEHOs were also responsible for using the megaphone to deliver messages recorded on a USB flash drive. Supportive supervision was provided to all CEHOs and model fathers four times during the intervention period.

### Participants and sample size

The sampling procedure for the initial study, from which the participants in the follow-on study were recruited, has been described elsewhere [28]. Briefly, the districts were selected because they had high prevalence of childhood stunting and poverty. Administrative cells, which are the smallest administrative units, containing 5–7 villages, were randomly assigned to intervention or control. Fathers in households enrolled in the initial study were eligible for participation in the follow-on study if they were ≥ 18 years, the household still owned or kept a cow, and the father was living in the household with his family. For the follow-on study, we included fathers from all study arms of the initial study in order to achieve the target sample size because many fathers had moved to other districts for work, some families had moved out of the district, and some families’ cows had died. All fathers enrolled in the follow-on study had an opportunity to hear SBCC messages as part of the initial study and to participate in the follow-on intervention targeted at fathers.

The survey sample size for the follow-on study was based on comparisons of proportions in one sample. Assuming sample estimates were 20% at baseline and would increase 10 percentage points to 30% by endline, a sample of N = 137 at 80% power and alpha = 0.05 was required. We added 10% to the sample size to account for attrition, giving a total sample size of N = 151.

At endline, we conducted separate focus group discussions (FGDs) with fathers (four FGDs, total of 24 fathers) and mothers (four FGDs, total of 24 mothers) and in-depth interviews (IDIs) with CEHOs (N = 6) and model fathers (N = 6). We chose these methods to gather perspectives from a variety of participants and implementers and to ensure that we could achieve data saturation [30]. Fathers were eligible to enroll in FGDs if they participated in the baseline for the follow-on study. Mothers were eligible to enroll in FGDs if their husbands participated in the baseline for the follow-on study and they themselves had participated in some aspect of the SBCC intervention for the follow-on study. CEHOs and model fathers were eligible to participate in IDIs, if they had been trained and had implemented the SBCC intervention that was part of the follow-on study.

### Data collection

Data were collected during or immediately following the short rainy season, which is typically from October to December, when cow milk production is high. Baseline quantitative survey data was collected from December 2020 to January 2021, and endline survey data was collected from October to November 2021. The enumerators entered the data on tablets with the questionnaire programmed in KoBoToolbox. The questionnaire was developed in English and translated into Kinyarwanda. It included questions on frequency of child milk and other ASF consumption in the last 7 days; fathers’ knowledge of ASFs, awareness of key nutrition practices, and support for children’s milk and other ASF consumption; and decision-making related to household ASF use. Fathers were also asked about demographics at baseline and intervention exposure at endline.

Endline qualitative data were collected by a separate team of trained data collectors in October and November 2021. The question guides for fathers and mothers asked about different components of the SBCC intervention and their experiences participating in them, household decision-making related to milk use, and ongoing barriers to ASF consumption. The question guide for CEHOs and model fathers included questions about different components of the SBCC intervention, their experiences implementing them, and their recommendations for improving the intervention. The guides were developed in English and translated into Kinyarwanda. FGDs and IDIs were digitally recorded, and the recordings were transcribed and translated into English.

Ethical approval for the study was obtained from the University of Rwanda’s College of Medicine and Health Sciences institutional review board (275/CMHS IRB/2020) and ILRI’s research ethics committee (ILRI-IREC2020-34). All participants provided written informed consent. Additional information regarding the ethical, cultural, and scientific considerations specific to inclusivity in global research is included in **S2 File**.

### Measures

Outcome variables were measured at baseline and endline. An ASF frequency questionnaire was used to measure children’s consumption of milk and other ASFs during the last 7 days, which were considered the main outcome variables. Fathers reported the number of times their child ate each type of ASF in the last 7 days in four categories (zero, one, two to four, or five or more times), which were dichotomized for the analysis (1 = consumed two or more times in last week, 0 = consumed fewer than two times in last week). Consumption of milk and other frequently consumed ASFs (fish, eggs, beef) were calculated as separate outcome variables, and a combined ASF consumption variable was created for all ASFs (cow milk, cow meat (beef), cow organs, goat meat, goat organs, sheep meat, sheep organs, pig meat, chicken meat, eggs, and fish), including those that were less frequently consumed. For each type of ASF consumed by children in the last 7 days, fathers were asked its source, whether the ASF was purchased, from own production, or a gift.

Fathers’ knowledge, awareness, and support for ASF consumption and household decision-making variables were considered secondary outcomes. Knowledge and support questions were asked without providing response options, whereas awareness was measured by asking if the father had ever heard the key messages. Knowledge questions were asked before awareness questions. Fathers’ knowledge was measured using four questions, which were dichotomized (1 = correct response, 0 = incorrect response) and summed to give a knowledge score ranging from 0 to 4. Awareness was measured using three questions, which were summed to give an awareness score ranging from 0 to 3. Fathers’ support for their children’s milk consumption and for their children’s other ASF consumption each contained four types of support, which were summed for milk and other ASFs separately and then dichotomized (1 = two or more supportive actions, 0 = one supportive action) for analysis. Decision-making about the use of home-produced milk was dichotomized (1 = decision-making done jointly by husband and wife, 0 = decision-making not done jointly) for the analysis. Socioeconomic characteristics, including number of household members, number of children < 5 years in the household, fathers’ age, fathers’ years of education, fathers’ main occupation, and marital status, were measured at baseline. Fathers’ exposure to different components of the intervention was measured at endline.

#### Data analysis

The survey analysis was performed using StataMP (version 17.0). Longitudinal fixed effects logistic or linear regression models with robust standard errors to account for clustering at the level of the administrative cell were used to assess changes in children’s milk and ASF consumption and fathers’ nutrition-related knowledge, awareness, and supportive actions. Models were adjusted for fathers’ age, education, and occupation, which may confound changes in the outcomes. To limit the number of statistical tests in the analysis, tests were performed only for the children’s milk and ASF consumption variables; composite knowledge and awareness scores; dichotomized two or more supportive actions variables; and the joint household decision-making variable.

FGDs and IDIs were analyzed using qualitative content analysis methods [31]. Four research assistants developed codebooks of deductive codes based on the question guides and coded the transcripts in Excel. The codes were grouped into themes, and the research assistants prepared narrative summaries for each theme. Results from the qualitative data were triangulated with survey findings, where appropriate, and supported with illustrative quotations.

## Results

Of the 151 fathers enrolled in the study, two were lost to follow-up. Therefore, the analysis includes 149 fathers who completed surveys at baseline and endline.

### Participant characteristics

Fathers who participated in the surveys lived in households that had received a cow 5.8 years ago on average (**Table 1**). Their households had an average of 6.1 members, including 1.4 children under 5 years of age. Fathers were 41.1 years of age on average and had a mean of 4.2 years of education. The majority of fathers worked as farmers and all of them were married.

**Table 1:** Father and household characteristics at baseline (N = 149).

	Baseline	
	%/Mean	n/SD
Years since receiving the Girinka cow (mean, SD)	5.8	3.1
Cow is alive (%, n)	98.7	149
Number of household members (mean, SD)	6.1	1.6
Number of children < 5 years (mean, SD)	1.4	0.5
Father’s age in years (mean, SD)	41.1	8.9
Father’s years of education (mean, SD)	4.2	3.0
Father’s main occupation (%, n)		
Farmer	88.6	132
Salaried employee	3.4	5
Small trader/self-employment	4.7	7
Jobless	2.0	3
Other	1.3	2
Father is married (%, n)	100.0	151

FGD participants’ mean age in years was 36.6 for mothers and 40.5 for fathers. FGD participants were from households with an average of two children under 5 years of age. Each household owned one milking cow from the Girinka program. IDIs with CEHO were conducted with four male and two female CEHOs. Their mean age in years was 39.8 for men and 40.5 for women. The mean age of model fathers interviewed was 42.7 years.

### Survey results

#### Changes in children’s ASF consumption

The ASFs most frequently consumed by children under 5 years were milk, fish, eggs, and beef (**Table 2**). The percentage of children who consumed any ASFs two or more times in the last 7 days increased from baseline to endline (76.5% to 92.6%). The percentage of children who consumed milk, eggs, and beef two or more times in the last 7 days also increased from baseline to endline, while the percentage who consumed fish did not change. Similarly, children had increased odds of consuming ASFs overall (odds ratio 4.9, 95% confidence interval 1.9, 12.3) and milk, eggs, and beef, in particular, two or more times in the last week.

**Table 2:** Children’s consumption of ASFs two or more times in the last 7 days (N = 149).

	Baseline		Endline		pp difference	OR^1^	95% CI
	%	n	%	n			
Any type of ASF^2^	76.5	114	92.6	138	16.1	4.9	1.9, 12.3
Milk	59.1	88	81.2	121	22.1	3.6	2.0, 6.4
Fish	40.9	61	40.9	61	0.0	1.0	0.6, 1.7
Eggs	22.8	34	53.0	79	30.2	4.0	2.3, 7.0
Beef	2.0	3	11.4	17	9.4	6.5	1.7, 24.0

ASF, animal source food; CI, confidence interval; OR, odds ratio; pp, percentage point

^1^Analyses account for clustering at the level of the cell using robust standard errors and adjust for father’s age, education, and occupation.

^2^Any type of ASF includes cow milk, cow meat (beef), cow organs, goat meat, goat organs, sheep meat, sheep organs, pig meat, chicken meat, eggs, and fish. Most of these types of ASF were rarely consumed. Only the ASFs that were more commonly consumed are shown in separate rows.

Sources of ASFs consumed by children in the last 7 days are shown in **S1 Table.** At endline, milk was sourced by purchasing (51.1%), own production (48.1%), or gift (0.8%); eggs were sourced by purchasing (30.7%) or own production (69.3%); and fish and beef were obtained by purchasing (100.0%). Sourcing of eggs from own production increased from baseline to endline (47.8% to 69.3%, *P*<0.05). No change in sourcing of other ASFs was detected.

#### Changes in fathers’ knowledge and awareness of ASFs

Fathers’ mean ASF knowledge score increased from baseline to endline (mean difference of 1.2 out of 4 items, *P*<0.001) (**Table 3**). Of the four items in the ASF knowledge score, the percentage of fathers who could independently name at least one ASF as a food young children need to grow and develop their brains, knew that children should start to receive cow milk at 12 months of age, and knew that children should start to receive ASFs other than cow milk at 6 months increased from baseline to endline, with especially large increases in the knowledge variables related to timing of introduction of milk and other ASFs. The percentage of fathers who knew that a child should drink 1 cup or more of milk per day increased only slightly from baseline to endline. Fathers’ mean ASF awareness score increased from baseline to endline (mean difference of 0.5 out of 3 items, *P*<0.001). Of the three items in the awareness score, the percentage of fathers who had ever heard each of the project’s key ASF consumption messages increased from baseline to endline.

**Table 3:** Fathers’ knowledge and awareness of ASFs (N = 149)^1^.

	Baseline		Endline		Mean difference	*P*-value^2^

KNOWLEDGE	Mean	SD	Mean	SD		
ASF knowledge score (0–4)	2.3	0.8	3.5	0.7	1.2	<0.001
	**%**	**n**	**%**	**n**	**pp difference**	
At least one ASF named as a food young children need to grow and develop their brains	84.6	126	98.0	146	13.4	
Child should drink 1 cup or more of milk per day	79.5	116	83.9	125	4.4	
Child should start to receive cow milk at 12 months of age	40.9	61	87.3	130	46.4	
Child should start to receive ASFs other than cow milk at 6 months of age	21.5	32	84.6	126	63.1	
**AWARENESS**	**Mean**	**SD**	**Mean**	**SD**	**Mean difference**	
ASF awareness score (0–3)	2.5	0.9	3.0	0.3	0.5	<0.001
	**%**	**n**	**%**	**n**	**pp difference**	
Ever heard about feeding the child ASFs	91.3	136	99.3	148	8.0	
Ever heard about feeding the child one cup of milk per day	79.9	119	98.7	147	18.8	
Ever heard about starting to feed the child cow milk at 12 months	79.9	119	97.3	145	17.4	

ASF, animal source food; pp, percentage point; SD, standard deviation

^1^Knowledge was measured without providing response options, and knowledge questions were asked before awareness questions. Awareness was measured by asking fathers if they had ever heard of specific key messages. Analyses account for clustering at the level of the cell using robust standard errors and adjust for father’s age, education, and occupation.

^2^Statistical tests were performed only for the knowledge and awareness scores, not for the individual items that were included in the scores.

#### Changes in fathers’ supportive actions for children’s milk and other ASF consumption and household decision-making about milk use

The majority of fathers offered one supportive action related to their children’s milk consumption, but the percentage of fathers who offered two or more supportive actions increased from baseline to endline (19.5% to 31.5%, *P* = 0.017) (**Table 4**). Fathers’ most common supportive actions for their children’s milk consumption were making sure milk from the household’s cow goes to children and giving their wives money to buy milk for children. These actions did not change from baseline to endline. However, the less common supportive actions of fathers’ purchasing milk for children and advising their wives to give children milk increased from baseline to endline. As with milk, the majority of fathers offered one supportive action for their children’s other ASF consumption, but the percentage of fathers who offered two supportive actions increased from baseline to endline (18.8% to 37.6%, *P*<0.001). Fathers’ most common action for non-milk ASF consumption was giving their wives money to buy ASFs for children, which decreased from baseline to endline. Less common supportive actions, including making sure ASFs from own production go to children, fathers’ purchasing ASFs for children, and advising their wives to give children ASFs increased from baseline to endline. The percentage of fathers reporting that decision-making about use of home-produced milk is made jointly by husband and wife increased from baseline to endline (64.4% to 77.9%, *P* = 0.002).

**Table 4:** Fathers’ support for milk and ASF consumption by children and household decision-making about use of home-produced milk (N = 149)^1^.

	Baseline			Endline		pp difference	P-value^2^

	%	n	%		n		
Fathers reported two or more supportive actions for children’s milk consumption	19.5	29	31.5		47	12.0	0.017
Make sure milk from household cow goes to children	59.5	88	55.7		83	−3.8	
Give wife money to buy milk for children	39.2	58	38.3		57	−0.9	
Purchase milk for children myself	11.5	17	23.5		35	12.0	
Advise wife to give children milk	6.1	9	16.1		24	10.0	
Fathers reported two or more supportive actions for children’s consumption of ASFs other than milk	18.8	28	37.6		56	18.8	<0.001
Make sure ASFs from own production go to children	24.3	36	46.3		69	22.0	
Give wife money to buy ASFs for children	71.6	106	53.7		80	−17.9	
Purchase ASFs for children myself	13.5	20	26.2		39	12.7	
Advise wife to give children ASFs	3.4	5	16.1		24	12.7	
Decision-making about use of home-produced milk is done jointly by father and mother	64.4	96	77.9		116	13.5	0.002

ASF, animal source food; pp, percentage point; SD, standard deviation

^1^Analyses account for clustering at the level of the cell using robust standard errors and adjust for father’s age, education, and occupation.

^2^Statistical tests were performed only for the summary supportive actions variables (fathers reporting two or more supportive actions) and for the joint household decision-making variable.

#### Fathers’ exposure to the intervention

Nearly all fathers (98%) had heard about ASFs through community-based meetings where milk or other ASFs were discussed in the last 4 months (**Table 5**). Most fathers (86%) reported that model fathers trained by the project were the leaders of the meetings where they heard about ASFs. Nearly all model fathers used the SBCC materials developed by the project, with the leaflet (94%) and poster (55%) most frequently used. Few fathers (2%) heard the megaphone messages about ASFs.

**Table 5:** Fathers’ exposure to the intervention (N = 149).

	Endline	
	%	n
Heard about ASFs through community-based meetings where milk or other ASFs were discussed	98.0	146
Person giving information about milk or other ASFs		
Model father	85.6	125
CHW	16.4	24
CEHO	4.8	7
Community leader	1.4	2
Agricultural extension agent	0.7	1
Community volunteer	2.1	3
Person used SBCC materials	98.6	144
Type of materials used		
Leaflet	93.8	135
Poster	54.9	79
Megaphone messages	2.1	3
Messages heard		
Importance of ASFs for children	98.0	143
Children should drink one cup of milk per day	99.3	143
Start giving the child milk at 12 months	99.3	144
Received text messages about milk or other ASFs	74.7	109
Messages received by text		
Importance of ASFs for children	96.2	102
Children should drink one cup of milk per day	97.2	104
Start giving the child milk at 12 months	99.1	108

ASF, animal source food; CEHO, community extension and health officer; CHW, community health worker; SBCC, social and behavior change communication

Three-quarters of fathers reported that they received text messages about milk or other ASFs. More than 95% of fathers who participated in meetings or received text messages about milk or other ASFs recalled the three key messages about giving ASFs to children.

### Qualitative results

Several key themes were identified in the FGD and IDI data. They are described here, and illustrative quotations are shown in **Table 6**. All fathers participating in the FGDs in both districts reported that they received messages on ASFs, milk consumption, and household nutrition during meetings with model fathers. Most fathers reported that meetings with model fathers took place at their sector offices. Most mothers explained that they heard about the fathers’ intervention messages from their husbands after the men had participated in the meetings held by model fathers. Some also said that their husbands shared the text messages they received about the importance of ASF and milk consumption for family nutrition.

**Table 6:** Key themes and illustrative quotations from qualitative data collected from fathers, mothers, model fathers, and CEHOs.

Key themes	Illustrative quotations
Selection of less expensive ASFs to overcome the financial barrier to ASF consumption	“In general, our barrier is poverty because we are not able to purchase some ASFs every day. We learned from the model fathers that we should try to feed our children cow meat, chicken meat, etc. But they also told us to replace them with small fish and eggs that are affordable for everyone. So, this gave us inspiration to try, knowing that we didn’t have to spend too much money and can still give our children the best of what they need.” —Father, 54 years, Ruhango “Since our husbands got trained, they now also care about making sure our children eat animal proteins, such as small fish and eggs. I would say that even if cow meat is expensive, I discuss with my husband and decide to purchase it sometimes in order to improve our household’s nutrition.” —Mother, 43 years, Ruhango
Increase in fathers’ knowledge about ASFs	“I liked that I gained knowledge about balanced diets for my children and when they can start to drink milk. Before I did not know they should wait until one year old to drink cow’s milk, but that my small child can eat eggs already at six months. This is very important knowledge to gain. My wife hears this from the community health worker, but now I also know this to be true.” —Father, 48 years, Nyabihu
Improvements in joint decision-making by fathers and mothers about use of home-produced milk	“Before I was selling the milk from my cow. My wife and the CHW told me to stop selling all of the milk, but I did not listen to them at that time. After being trained by the model father, a man that I know and respect, my behavior has changed. He told me the same things that the CHW had said. I understood that milk is very important for my children to grow, and it is my responsibility to support their health. Now I keep some milk for drinking at home.” —Father, 33 years, Ruhango
Importance of monthly community-based meetings	“I liked how the model father encouraged us [during the meetings] to support our wives in feeding our children flesh foods and cow’s milk. This is a good example to follow, and I want to be an example for my neighbors, too.” —Father, 36 years, Nyabihu “There are some men called model fathers who trained our husbands about feeding animal source foods and cow’s milk to our children to improve [their] nutrition. And then our husbands shared with us the skills they got from those meetings.” —Mother, 30 years, Ruhango
SBCC materials were acceptable and provided clear actions and timely reminders for fathers	“I liked the images showing cooperation between a man and his spouse in preparing foods. Before I did not help my wife in doing household chores, but today I try to help her, even if our neighbors called me ‘a tame man,’ but this makes me proud of supporting my wife and I want to be an example to follow for my son and other fathers in my community.” —Model Father, 33 years, Ruhango “For the poster, I liked the way it was designed with clear and understandable messages explaining how a father can contribute to his children’s wellbeing. The images show exactly what the father can do to support and be part of the solution to fight against malnutrition in the household.” —CEHO, 37 years, Nyabihu “As I am focusing on milking, the text messages remind me not to sell all the milk but to make sure I keep some and feed it to my family. This helps my family to grow strong and healthy.” —Father, 38 years, Nyabihu
Recommendations	“In the beginning, it was not easy to get a place at the sector to meet together with intervention fathers. The sector officials didn’t know about the meetings and were not happy to support us. Once Three Stones introduced the project to them, then they were more helpful. It is better to share letters of collaboration before the project starts and to involve these district officials in the sessions.” —Model Father, 55 years, Ruhango

#### Selection of less expensive ASFs to overcome the financial barrier to ASF consumption

All fathers in FGDs mentioned that lack of financial means to purchase ASFs, especially flesh foods, is the main barrier to consumption of ASFs in their household. However, with encouragement from model fathers and reminders through text messages, fathers explained that they tried their best to find more affordable ASFs for their young children, such as small fish and eggs. Mothers made similar comments about fathers seeking less expensive ASFs for their children and discussing with their husbands which types of ASFs to buy.

#### Increase in fathers’ knowledge of ASFs

Most fathers in FGDs discussed the relevance of the messages received from model fathers, which supported improving their knowledge on the appropriate age to introduce ASFs to young children. Fathers in both districts expressed that the knowledge they gained to improve household nutrition was the most important aspect of the messages they received through the program.

#### Improvements in joint decision-making by fathers and mothers about use of home-produced milk

Fathers said the second most important aspect of the program was improvements in joint decision-making with their wives about household milk use. The majority of fathers in the FGDs explained that they were inspired by the model fathers to jointly discuss with their wives how much cow’s milk from daily milking to keep for household consumption. Fathers said that they were encouraged to do so, as they understood the benefits of ASFs to help their children grow well.

#### Importance of monthly community-based meetings

Fathers liked the monthly meetings that were facilitated by the model fathers because they encouraged the family to eat and buy ASFs that were accessible to them. They were inspired by the model fathers’ actions as examples in their own communities. Most mothers stated that they noticed positive changes in their husbands’ actions after attending the sessions with model fathers. Mothers noted that their husbands shared the messages that they had heard and discussed how they could put these teachings into action as parents.

CEHOs and model fathers explained that monthly sessions were most appreciated as they brought a diverse group of people together to share the messages more widely. They found the meetings to be engaging and considered them a supportive setting to discuss experiences with other men in their communities. A few CEHOs and model fathers said that they initially faced challenges in gaining support from community leaders to agree to venues and support to conduct activities.

#### SBCC materials were acceptable and provided clear actions and timely reminders for fathers

Almost all fathers in FGDs in both districts said that they related to the messages and images on the leaflet and poster shared by model fathers during monthly meetings. They found the images inspirational and appreciated that they showed fathers happy and working with their wives. Receiving text messages helped to reinforce the messages on the leaflet and acted as a reminder for the fathers at critical times.

Most CEHOs and model fathers agreed that the messages were relevant and relayed important and useful information to support adoption of optimal young child feeding practices, recommended food safety and hygiene practices, and family cohesion. CEHOs said that the educational materials and key messages provided clear, short, and understandable messages that called fathers to action. Model fathers expressed that the use of positive parenting images inspired them and other fathers and showed clear representations of the key messages that were understandable for those who could not read.

#### Recommendations for improved implementation

Model fathers requested additional training on facilitation skills and suggested that follow-up sessions at household level would provide the opportunity to observe men putting the messages learned into action and would highlight where more support is needed. CEHOs and model fathers suggested that future interventions should train community leaders at the outset to increase their support and collaboration by making them aware of the importance of the community-based meetings. Model fathers and CEHOs agreed that more model fathers, community volunteers, community health workers, and CEHOs should be trained, and more leaflets produced for wider implementation within the existing districts and for further scale-up. CEHOs also suggested that the megaphone blasts should include testimonies from mothers.

## Discussion

This study found that an SBCC intervention encouraging fathers in livestock-keeping households to support child nutrition increased the percentage of children who more frequently consumed ASFs, specifically milk, eggs, and beef. ASFs are important sources of protein, iron, zinc, vitamin A, and other micronutrients [1], and consumption of ASFs contributes to child growth and development [2]. In this study, children’s consumption varied widely by type of ASF. The percentage of children who frequently consumed milk or eggs increased substantially from baseline to endline. Milk was the most commonly consumed ASF by children at baseline and endline. Approximately half of the milk consumed by children was from own production at both time points, indicating that households continued to allocate the milk they produced to their children. There was a small increase in purchasing milk from baseline to endline, most likely to cover low household milk production. The increase in egg consumption was mainly through increased use of eggs produced in the household. This finding is important, because the percentage of young children in Rwanda consuming eggs has been consistently very low (4.0% in 2014; 7.7% in 2019–2020) [32, 33], and this study shows that a large increase in the frequency of children’s eggs consumption is possible through own production, even without an accompanying poultry production intervention. Beef consumption also increased, but the percentage of children consuming beef at endline remained low due to high cost and the necessity of purchasing this type of ASF. Fish consumption by children was already common at baseline and did not change significantly from baseline to endline. Fish are not locally produced in the two study districts, but small dried fish are a relatively low-cost and readily available source of ASFs in these locales.

The initial study found smaller percentage point changes in children’s milk consumption than the follow-on study but showed that mothers’ intervention exposure was associated with the frequency of ASF consumption [28]. It is possible that effects were larger in the follow-on study because of increases in joint decision-making about use of home-produced milk or increases in fathers’ support for their children’s milk and other ASF consumption. However, the differences in the results of the follow-on and initial studies may be an artifact of the different study designs.

This study adds to the limited literature on SBCC interventions to engage fathers in child nutrition by providing evidence on the impacts of a multi-component intervention on child ASF consumption and fathers’ knowledge and support through quantitative data collected from fathers. To our knowledge, only one other study, in Nigeria, has used multiple channels to reach fathers on child feeding and measured changes in fathers’ knowledge and support [22]. Other programs in Bangladesh and Ethiopia reached fathers to support child feeding practices within their households through multiple channels, including community mobilization activities, mass media, and, in some cases, individual interpersonal communication, but did not collect data from fathers [23, 25]. Several other studies used a single channel to reach fathers, including peer education in community-based groups for fathers in Kenya, Malawi, and Haiti [6, 19, 24, 34, 35], community mobilization activities in Bangladesh [26], home visits with separate meetings for fathers in Uganda [36], and separate mobile health (mHealth) voice messaging for fathers and mothers in Senegal [21]. Of these, only the studies in Kenya and Senegal collected data on changes in fathers’ knowledge or support [19, 21].

Health-related knowledge and awareness are considered prerequisites for behavior change [37, 38]. Although most child nutrition studies measure maternal knowledge, two studies in Ethiopia demonstrated that fathers’ knowledge of child feeding is associated with child dietary diversity [39, 40]. A study in Senegal showed that an mHealth SBCC intervention can improve fathers’ nutrition knowledge [21]. Our study also showed that an intervention engaging fathers substantially improved fathers’ knowledge and awareness related to ASFs. The largest increases in fathers’ knowledge were on the timing of when to introduce milk and other ASFs. As with mothers in the initial study, we found that most fathers in the follow-on study already knew that children should drink 1 cup of milk per day because of the Rwandan government’s One Cup of Milk per Child campaign for school children [41].

Social support from fathers for child feeding practices is often instrumental in ensuring that mothers are able to carry out optimal practices or that mothers and fathers work together to implement them [19, 42], and interventions targeted at fathers can be effective at increasing their social support for child feeding practices [19]. Our study showed that the SBCC intervention increased fathers’ support for children’s ASF consumption and also increased joint decision-making by husband and wife about use of home-produced milk. We found that more than half of fathers already made sure their children were given milk from the family’s cow and this did not change. For other ASFs, we observed a large increase in the number of fathers who made sure their own production, most likely of eggs, went to children. More fathers also purchased milk and other ASFs, and more of them advised their wives to give children milk and ASFs. Despite these increases, at endline, only about one-quarter of fathers purchased milk or ASFs, indicating that further increases could be achieved. We also observed that the percentage of fathers who gave their wives money to buy ASFs for children decreased. This may have been because children were getting ASFs from home production or because fathers were purchasing the ASFs themselves.

It is important for evaluations to report on participants’ intervention exposure to make the case that the intervention led to the observed outcomes; however, few other intervention studies have reported fathers’ intervention exposures. Fathers’ exposure to the intervention components in this study was quite high for all elements except megaphone blasts and was verified by monitoring data collected during implementation. A higher percentage of fathers in this study recalled receiving nutrition-related text messages than in studies in Senegal and Nigeria [21, 22]. More of them also reported exposure to community-level activities in this study than in the study in Nigeria [22]. It is possible that fathers in our study were primed to listen to and implement the messages, because their families had already been part of the initial study.

Fathers in our study liked having role models (e.g., model fathers) whose practices they could emulate and who were trusted sources of information. This aligns with findings from a study in Kenya, which used peer educators to lead fathers’ groups [24]. Fathers in our study appreciated images that showed men and women collaborating and messages about specific actions fathers can take to support child nutrition in their households, which was consistent with learnings from large-scale infant and young child feeding SBCC programs that had components targeted at fathers [43].

This study had several strengths and limitations. The strengths were use of formative research to design the SBCC intervention and close collaboration on design and implementation with national- and district-level government agencies. The main limitations were the lack of a control group, the possibility of social desirability bias in the survey and qualitative responses, and COVID-related disruptions. It was not possible to have a control group, because all households in the two districts that had a child in the appropriate age range and that were Girinka participants or Girinka-eligible had already been enrolled in the initial study; therefore, we chose a longitudinal pre/post design. The changes found in this study are likely attributable to the intervention, because no other programs targeting ASF messages to fathers were implemented in these areas. Mothers in FGDs reported hearing ASF messages during community meetings for women, which indicates sustainability of our initial study intervention but also suggests that the follow-on study may be at least partially detecting effects of the initial study targeted at mothers. All primary and secondary outcomes were based on self-report by fathers, which may be subject to bias. Knowledge and support questions were open-ended, which helps to limit response bias. The percentage of children reported to have consumed different ASFs varied widely by type of ASF, suggesting that fathers were being truthful. Self-reported intervention exposure data was corroborated by monitoring data collected during implementation. Our qualitative data asking about opinions on the intervention also may have been subject to social desirability bias. To counterbalance that possibility, we asked about and presented barriers to implementation and challenges with carrying out the recommendations. Given that not all children in the study consumed ASFs at endline, issues related to the cost and availability of ASFs remain and could not be addressed by a behavior change intervention alone.

In conclusion, this study showed that a low-cost SBCC intervention that reached out to fathers improved child feeding practices, increased fathers’ knowledge of and support for child feeding, and increased joint decision-making by fathers and mothers about the use of home-produced foods. Our findings suggest that nutrition SBCC interventions specifically for fathers can be beneficial, but several gaps remain. More studies and programs that involve fathers should collect data from fathers, so that we can better understand changes in fathers’ knowledge and practices and how they related to nutrition outcomes. Work by Ambikapathi et al. [39] indicates that men’s and women’s knowledge are additive, but research is needed to use that information to develop impactful programs. Program implementers need evidence to help them determine whether child nutrition interventions should have a separate strategy for fathers, be combined with women’s empowerment interventions, use a gender transformative approach, or try to incrementally shift gender norms. Finally, effective child nutrition programs that involve fathers should document their intervention strategies and facilitators and barriers to implementation. Such information can be used to develop best practices for engaging fathers in child nutrition.

## Supporting information

S1 File Messages and images for engaging fathers to support child nutrition in Rwanda. (DOCX)

S2 File Inclusivity in global research checklist. (DOCX)

S1 Table Sources of ASFs consumed by children in the past 7 days. (DOCX)
