# Supplementary material for: Engaging fathers to support child nutrition increases frequency of children’s animal source food consumption in Rwanda
Source: PLoS One. 2023 Apr 7;18(4):e0283813. doi: 10.1371/journal.pone.0283813 (PMC10081762; doi:10.1371/journal.pone.0283813)
Supplement: S1 File — (DOCX) [file pone.0283813.s001.docx]

**S1. Messages and images for engaging fathers to support child nutrition in Rwanda**

**S1A. Titles, messages, and images for posters and leaflets^1^**

| Title | Message | Image |
| --- | --- | --- |
| **Milk is valuable for your family’s health!** | Not enough milk for drinking and to sell? Try keeping the evening milk produced by your cow for your family and use the morning milk for sale. Making sure your pregnant or lactating wife and young children drink cow’s milk will help support your children's growth and development! | 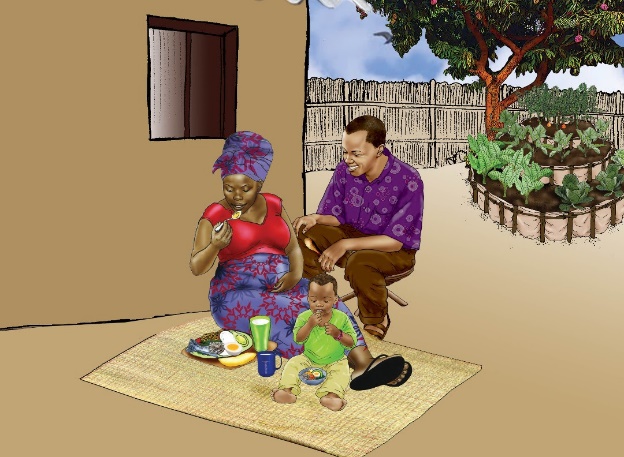 |
| **Avoid malnutrition in your family.** | Did you know that children can start drinking cow’s milk from 12 months of age? They can! Start feeding boiled cow’s milk at 12 months to prevent malnutrition and help your children grow strong while continuing to breastfeed and give other complementary foods. | **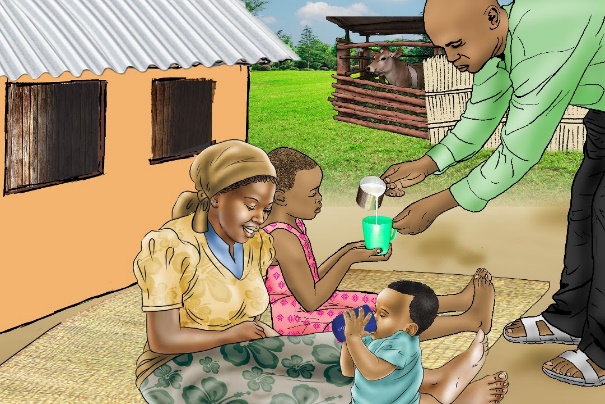** |
| **Be a nutrition champion!** | Malnutrition can have harmful, long-lasting effects on your family. Avoid this by making sure milk from your cow goes to your children and pregnant or lactating wives. From 6 months old, start giving children small fish, eggs, chicken, and meat every day to avoid malnutrition. Make animal source foods easy for young children to eat by mashing eggs or chopping fish or meat into very small pieces. That way, you are helping them to grow healthy and strong! | 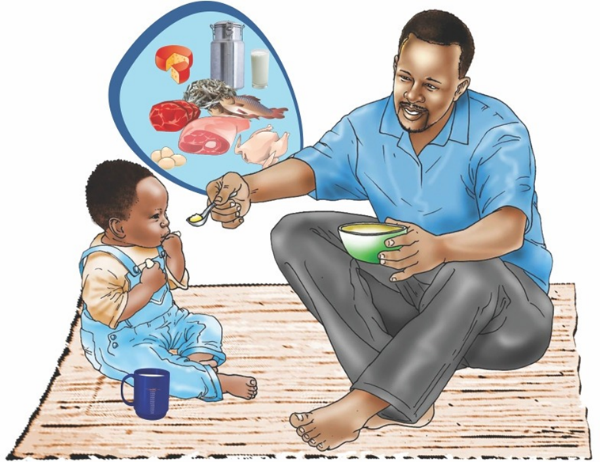 |
| **Grow a strong family.** | Make it your responsibility to ensure your family eats animal source foods or cow’s milk every day. This will help your family avoid visits to the doctor, participate in school, and have energy to do household chores. | 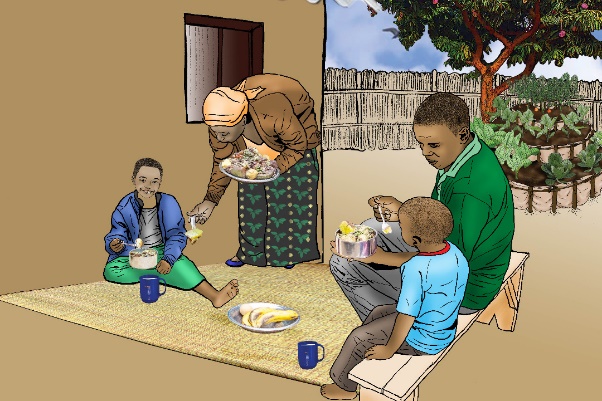 |
| **Cooking together builds a strong family!** | Cooking is an important job because it helps to keep your family be healthy and productive. Have your turn in the kitchen and discuss with your wife how you can include animal source foods in your family’s daily diet. Decide with your wife how to spend your income and allocate household resources, such as milk, for the family. | 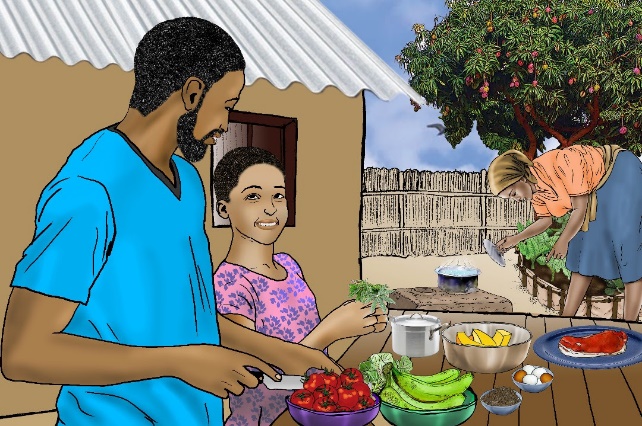 |
| **How to boil milk safely.** | To make sure you get the most out of your milk production, follow safe hygiene practices at home, such as boiling your raw milk before your family uses it. Boiled milk can be stored up to four hours at room temperature if covered, and five to seven days if refrigerated below 4°C. | **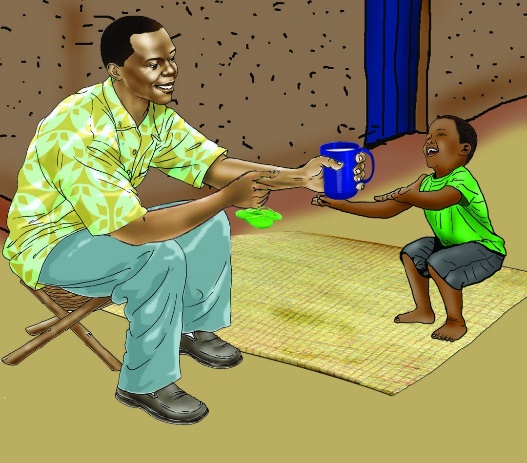** |
| **Make milk safe for drinking.** | 1. Remember to wash hands before food preparation, eating, and feeding your children. 2. Bring milk to a boiling point in a clean pot. 3. Store it in a stainless steel or metal container, such as a saucepan, after boiling. 4. Cover with a lid. 5. Allow to cool before drinking or feeding to your children within 4 hours. | 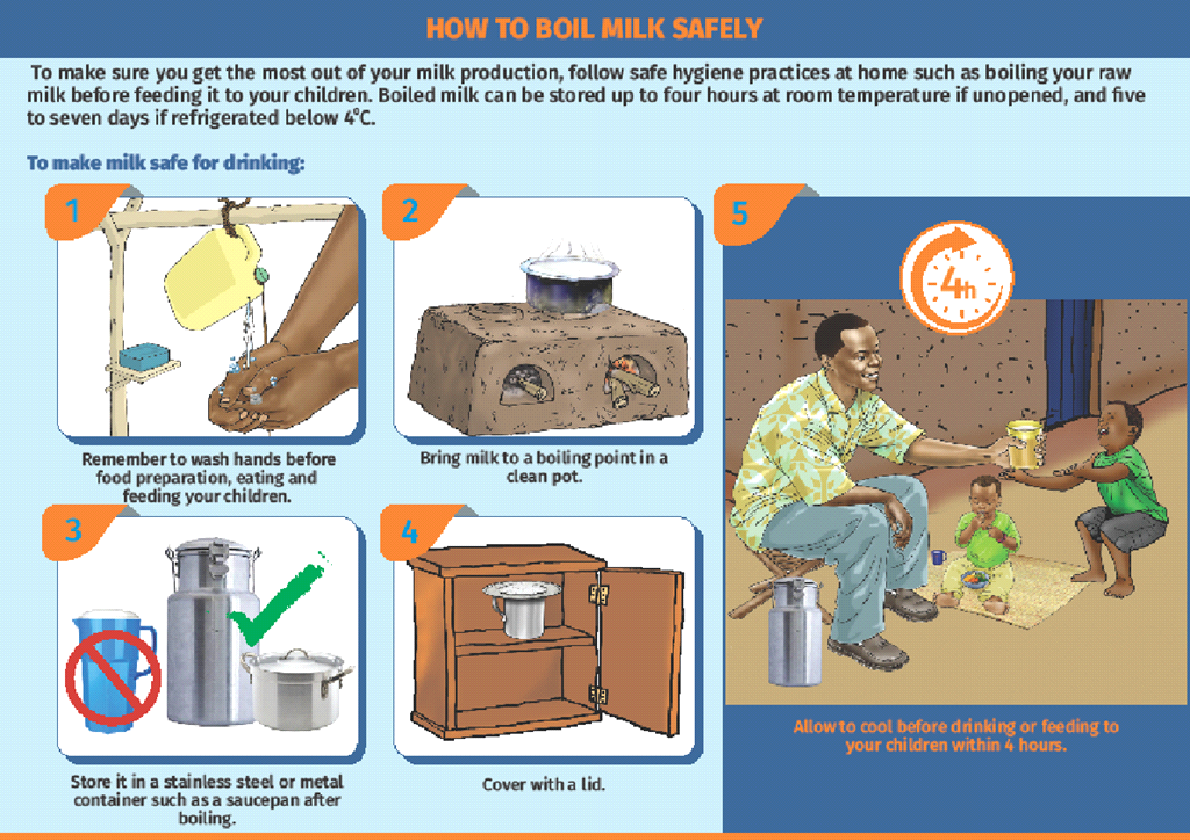 |

^1^Three Stones International adapted these images accessed from the USAID Advancing Nutrition-UNICEF IYCF Image Bank (iycf.advancingnutrition.org). Commercial use, redistribution, or selling of these images is prohibited.

**S1B. Megaphone Blast Testimonials**

“Men, don’t make the same mistake I did. Before I knew how important milk and animal source foods were for my children and family, I did not care about what they ate as long as they were not hungry. When I learned that my 1-year-old child was malnourished, I felt so ashamed. The village leader said I wasn’t providing well for my family. Then I learned through *Gabura Amata Mubyeyi* that milk and animal source foods, like small fish and eggs, are important for my children and my pregnant wife! Now I try give at least one cup of milk to each of my family every day. My children are no longer malnourished, and I am proud to say my family is healthy and prosperous.”

“Calling all fathers and husbands, let me tell you how important looking after the nutrition of your family is. I own a cow and used to sell all the milk every day. Sometimes I would spend that money on unimportant things, like beer. This meant my family did not have nutritious meals because we could not afford to buy meat or small fish. My children became malnourished, and I was scared for their life. I noticed that my neighbor’s children were always healthy and I asked him the secret. He said it was not secret and explained that he and his wife started feeding their child small fish, eggs, chicken, and meat every day when the baby was 6 months old. Then at 12 months old they also started giving the child cow's milk to avoid malnutrition. He also reminded me to support my wife to continue breastfeeding my child until at least 2 years!  I realized I had been wrong to sell all the milk because it was valuable for my family. Now I only sell the milk after everyone has one cup of milk to drink each day, and my wife and children are very happy and healthy!”

“As our village leader always says, “Many hands make light work,” and I agree! Working together with my wife and discussing important decisions, like how money should be spent and what healthy foods to purchase, or what crops to grow and how to share the household chores, helps to avoid conflict. A family that works together is a prosperous family. Be a good role model for your children by working as a team with your wife.”

“You know in Rwanda how much we love our cows, am I right? Well, we also love our milk, and I am sure all of you fathers out there will agree with me that not a drop should be wasted. Therefore, while you take good care of your cow by feeding her well to get regular milk production, also take care of the milk she produces. This is both valuable for your family's health and pockets. By boiling the fresh milk first, cooling and storing it in a stainless steel container like a cooking pot with a lid, you kill all the harmful bacteria that might make your children and family sick. Don’t let the milk be wasted and help to make your family strong and healthy.”
