# Supplementary material for: Engaging fathers to support child nutrition increases frequency of children’s animal source food consumption in Rwanda
Source: PLoS One. 2023 Apr 7;18(4):e0283813. doi: 10.1371/journal.pone.0283813 (PMC10081762; doi:10.1371/journal.pone.0283813)
Supplement: S1 Table — (DOCX) [file pone.0283813.s003.docx]

**S3. Sources of ASFs consumed by children in the last 7 days**

|  | **Purchase** | | | | **Own Production** | | | | **Gift** | | | |
| --- | --- | --- | --- | --- | --- | --- | --- | --- | --- | --- | --- | --- |
|  | **Baseline** | | **Endline** | | **Baseline** | | **Endline** | | **Baseline** | | **Endline** | |
|  | % | N | % | N | % | N | % | N | % | N | % | N |
| Milk | 44.2 | 46 | 51.1 | 68 | 53.9 | 56 | 48.1 | 64 | 1.9 | 2 | 0.8 | 1 |
| Fish | 100.0 | 66 | 100.0 | 72 | 0.0 | 0 | 0.0 | 0 | 0.0 | 0 | 0.0 | 0 |
| Eggs* | 51.1 | 24 | 30.7 | 27 | 46.8 | 22 | 69.3 | 61 | 2.1 | 1 | 0.0 | 0 |
| Beef | 100.0 | 21 | 100.0 | 48 | 0.0 | 0 | 0.0 | 0 | 0.0 | 0 | 0.0 | 0 |

**P*<0.05 for comparison between baseline and endline
